# The spatial econometrics of the coronavirus pandemic

**DOI:** 10.1007/s12076-020-00254-1

**Published:** 2020-08-01

**Authors:** Tamás Krisztin, Philipp Piribauer, Michael Wögerer

**Affiliations:** 1 International Institute for Applied Systems Analysis (IIASA), Schloßplatz 1, 2361 Laxenburg, Austria; 2 Austrian Institute of Economic Research (WIFO), Vienna, Austria

**Keywords:** Coronavirus COVID-19, Spatial econometrics, Spatial spillovers, Bayesian Markov-chain Monte Carlo (MCMC), C11, H12, I18, R10

## Abstract

In this paper we use spatial econometric specifications to model daily infection rates of COVID-19 across countries. Using recent advances in Bayesian spatial econometric techniques, we particularly focus on the time-dependent importance of alternative spatial linkage structures such as the number of flight connections, relationships in international trade, and common borders. The flexible model setup allows to study the intensity and type of spatial spillover structures over time. Our results show notable spatial spillover mechanisms in the early stages of the virus with international flight linkages as the main transmission channel. In later stages, our model shows a sharp drop in the intensity spatial spillovers due to national travel bans, indicating that travel restrictions led to a reduction of cross-country spillovers.

## Introduction

Are spatial econometric methods suitable to model the recent global spread of the coronavirus (COVID-19)? Overall, there is a vast literature on spatial data analysis with a rather heterogeneous treatment of spatial dependence and spillover structures (see, for example, Zoller 2004). Spatial econometric specifications (LeSage and Pace 2009) make the spatial dependence structures among the observations particularly explicit (Bivand et al. 2015). These approaches aim at highlighting the importance of directly accounting for spatial interdependencies among the observations under scrutiny and have recently gained momentum particularly in the regional science and economics literature. Spatial econometric specifications use so-called spatial weight matrices in order to augment standard classical linear model specifications by allowing for spatial spillovers among the observations (see, LeSage and Pace 2009; Anselin 2013). Multiple previous studies highlight the importance of spatial econometrics for capturing disease transmission pathways and network effects, as well as in quantifying the magnitude of spatial spillovers (see, e.g. Emch et al. 2012; Wang et al. 2015; Chagas et al. 2016).

For modelling the recent coronavirus pandemic, allowing for spatial dependence appears of predominant importance. In the beginning of the crisis (January 2020), the virus was often seen as a Chinese and later an Italian problem. However, due to the rapid spread of the virus across the globe, almost all Western countries reacted by employing drastic measures to contain or delay the further spread of the virus. These measures entail considerable restrictions in every day social and economic life. Most notable policy measures comprise closings of borders and general curfews to curb the spread. At the beginning of the outbreak, national linkages thus appear of particular importance to explain the spread across the globe.

In this paper we use spatial econometric frameworks to model COVID-19 infections across the globe. Our spatial econometric specification pays particular attention to different types of spatial dependence including information on geographic neighbourhood, travel linkages as well as trade ties. By using daily data on country-specific infections, we moreover allow the strength of spatial dependence to vary over time. Both features appear to be of particular importance for adequately modelling the spread of the virus.

## A spatial dynamic panel model

We make use of a spatial autoregressive (SAR) dynamic panel model by particularly focussing on time-dependent spatial dependence structures during the spread of the virus, which we aim to model for *N* countries over *T* days. The model can be written as follows:

2.1 $$\begin{aligned} \varvec{y}_t = \rho _t\varvec{W}(q_t) \varvec{y}_{t} + \varvec{\alpha } + \varvec{y}_{t-1}\beta + \varvec{\varepsilon }_t \end{aligned}$$

where $\varvec{y}_{t}$ is an $N \times 1$ vector of country-specific infections at time *t* ($t=1,\ldots ,T$), $\varvec{W}(q_t) \varvec{y}_{t}$ denotes the so-called spatial lag, and $\varvec{\alpha }$ contains country-specific trends. The $N \times 1$ vector of innovations $\varvec{\varepsilon }_t$ is assumed iid normal with zero mean and variance $\sigma ^2$.

Note that the spatial autoregressive term in (2.1) comprises a time-dependent $N\times N$ spatial weight matrix $\varvec{W}(q_t)$. The (scalar) parameter $\rho _t$ measures the strength of spatial autocorrelation and is also modelled time-variant. Positive (negative) values of $\rho _t$ indicate positive (negative) spatial autocorrelation, with sufficient stability condition $\rho _t \in (-\,1, 1)$ for all *t* (see LeSage and Pace 2009).

The spatial weight matrix $\varvec{W}(q_t)$ captures spatial linkages between countries *i* and *j* (with $i,j = 1,\ldots ,N$) at time *t*. $\varvec{W}(q_t)$ is non-negative and row-stochastic, with entries treated as known constants. Its typical element $\left[ \varvec{W}(q_t)\right] _{ij} = 0$ for $i = j$ and $\left[ \varvec{W}(q_t)\right] _{ij} > 0$, if there is a considered link between countries *i* and *j*. Moreover, $\left[ \varvec{W}(q_t)\right] _{ii} = 0$, as no region is considered to be its own neighbour.

The time-variant discrete parameter $q_t \in \{1,\ldots ,P\}$ governs the choice of a spatial weight matrix from *P* alternatives, eventually of different classes, and is to be estimated. In the spirit of work by Piribauer and Crespo Cuaresma (2016) and Fischer and LeSage (2015), such a specification allows to trace the nature of global spatial spillovers over time in a flexible way. Bayesian estimation techniques allow to efficiently deal with such flexible mixture specifications of spatial weight matrices. In addition to the time-variant spatial autoregressive parameter, the proposed model specification thus also allows to study the nature of spatial spillover processes over time.

In the spatial econometrics literature, spatial spillovers are defined as the impacts to a region’s outcome variable due to shocks in other regions. By reformulating Eq. (2.1) to its reduced form representation, $\varvec{y}_t = \left( \varvec{I}-\rho _t\varvec{W}(q_t)\right) ^{-1}( \varvec{\alpha } + \varvec{y}_{t-1}\beta + \varvec{\varepsilon }_t)$, the spatial multiplier matrix $\left( \varvec{I}-\rho _t\varvec{W}(q_t)\right) ^{-1}=\sum _{r=0}^\infty \rho _t^r\varvec{W}(q_t)^r$ for a given spatial weight matrix is governed by the spatial autoregressive parameter $\rho _t$ (for a thorough discussion, see LeSage and Pace 2009). By accounting for both spatial and non-spatial components, our proposed model thus allows to distinguish between intra- and cross-regional transmission processes.

Since country-specific infections are non-negative count data, one may argue that a spatial econometric specification for count data might be more suitable. However, in such cases explicit spatial autoregressive specifications are much more difficult to handle (see Bivand et al. 2014 or LeSage and Pace 2009). As a benchmark model, we therefore also apply a popular alternative in the spatial econometric literature put forward by Fischer et al. (2006), by using a Bayesian Poisson framework with an explicit spatial autoregressive error structure. This model (henceforth labelled Poisson spatial error model—Poisson SEM) can be written as follows:^1^

2.2 $$\begin{aligned} \varvec{y}_t&\sim {\mathcal {P}}(\varvec{\lambda }_t) \nonumber \\ \varvec{\lambda }_t&= \exp \left( \varvec{\alpha } + \varvec{y}_{t-1}\beta + \varvec{\nu }_t \right) , \end{aligned}$$

where ${\mathcal {P}}(\cdot )$ denotes the Poisson distribution and the $N \times 1$ vector $\varvec{\lambda }_t$ is the mean of the Poisson process. The $N \times 1$ vector $\varvec{\nu }_t$ captures country specific random effects. We follow work by LeSage et al. (2007) and introduce a source of spatial dependence via the random effects vector $\varvec{\nu }_t$, which is assumed to follow a first order spatial autoregressive process:

$$\begin{aligned} \varvec{\nu }_t&= \phi _t \varvec{W}(q_t) \varvec{\nu }_t + \varvec{\eta }_t \qquad \varvec{\eta }_t = {\mathcal {N}}(0,\tau _t^2 \varvec{I}). \end{aligned}$$

The scalar $\phi _t \in (-\,1,1)$ measures the strength of spatial autocorrelation at time *t*, and $\varvec{W}(q_t)$ is specified as above. The disturbance error vector $\varvec{\eta }_t$ is assumed to be iid normally distributed with zero mean and $\tau ^2$ variance.^2^

## Data, spatial weights and estimation

**Fig. 1:**
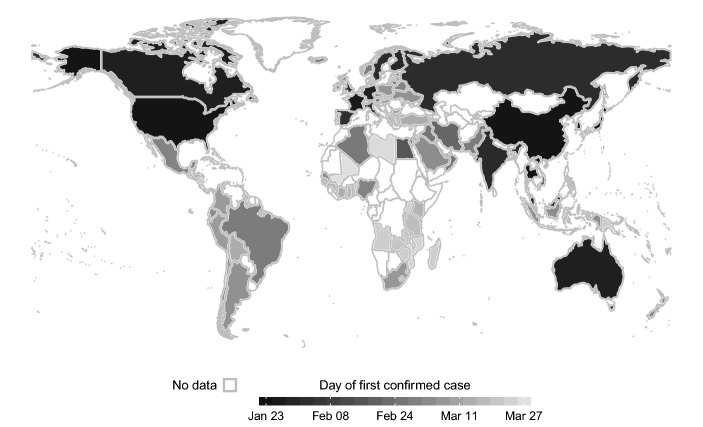
First confirmed cases by country

We make use of the COVID-19 database provided by the Johns Hopkins University (Dong et al. 2020). The data set contains information on daily case counts of confirmed infections for 99 countries. Our dependent variable is the logged daily number of confirmed cases per 100,000 inhabitants per country from January 23rd 2020 to March 28th 2020. Population data is obtained from the *World Bank* development indicators database. Figure 1 depicts a map of the countries in our sample along with the country-specific timing of the coronavirus outbreak.^3^

We allow for four alternative types of spatial weight matrices: first, the presence of *common borders* (Source: Eurostat). Second, the intensity of bilateral *flight connections*, measured in terms of the total number of weekly commercial flights between country pairs (Source: openflights.com). Third, *trade intensity*, using aggregate trade in 2010 USD to construct a *k*-nearest neighbour type spatial weight matrix, where the seven partners with highest aggregate trade value were considered (Source: *WITS* trade database).^4^ Finally, international agreements guaranteeing the *free movement of people*, where if a country pair has signed a treaty allowing for free movement of people, they are considered to be neighbours (Source: Krisztin and Fischer 2015).

For the SAR model in Eq. (2.1), estimation is carried out using well-known Bayesian Markov-chain Monte Carlo (MCMC) sampling techniques. For the Poisson SEM model in Eq. (2.2), we use the sampling algorithm laid out in Fischer et al. (2006) and Frühwirth-Schnatter et al. (2009). For both specifications we use rather non-informative Gaussian priors for the parameters $\varvec{\alpha }$ and $\beta$, with zero mean and a large variance of $10^8$. For $\sigma ^2$ and $\tau ^2_t$, we similarly elicit a common inverted gamma specification $\mathcal {IG}(0.001,0.001)$.^5^ For $\rho _t$ and $\phi _t$ we use a standard beta prior specification as suggested in LeSage and Pace (2009). For the choice of the alternative spatial weight matrices $q_t$, a non-informative uniform prior specification is used.

Sampling for the parameters $\varvec{\alpha }$, $\beta$, $\sigma ^2$, and $\tau ^2$ is done using standard conditional posteriors (see, LeSage and Pace 2009; Fischer et al. 2006). To account for the heterogeneity of $\rho _t$ and $\phi _t$, we use a sampling strategy discussed in LeSage and Chih (2018). Sampling for $q_t$ is discussed in Piribauer and Crespo Cuaresma (2016).^6^

## Results

**Fig. 2:**
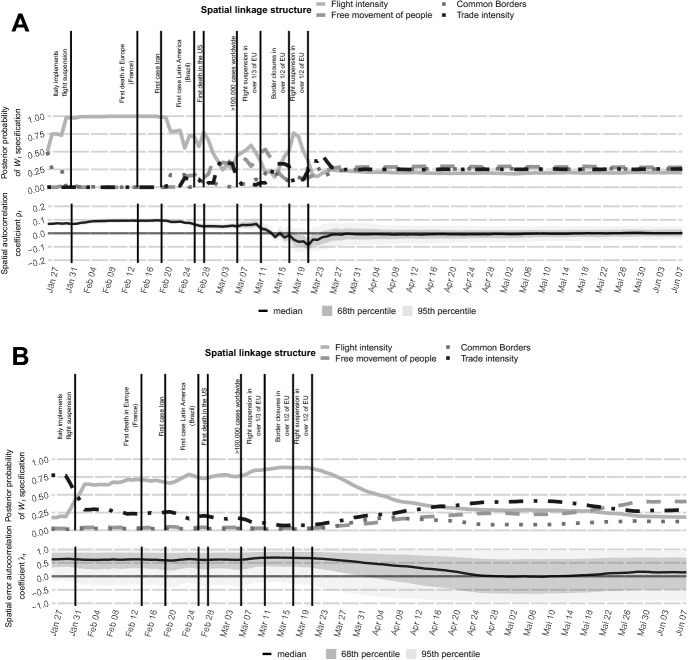
Posterior parameter estimates for the spatial dynamic panel SAR (**a**) and Poisson SEM (**b**) specifications. Top panels indicate posterior inclusion probability of spatial weight matrices over time. Bottom panels indicate the smoothed posterior median of the spatial autoregressive parameter $\rho _t$ and $\lambda _t$, respectively

We present the MCMC estimation results obtained from 20,000 posterior draws, where 10,000 were discarded as burn-ins.^7^ Estimation results are summarised in Fig. 2 and Table 1. Table 1 summarizes estimation results for the SAR and Poisson SEM specifications. The country-specific intercepts are excluded for the sake of brevity.

A first inspection reveals that the temporal autoregressive parameter $\beta$ is—as expected—statistically significant in both specifications. Forecasting our fitted SAR model results—on average across countries and time—in a doubling rate of confirmed infections every 4 days. The $R^2$ values of 0.905 (SAR) and 0.979 (Poisson SEM) indicate that the proposed models appear to fit the data very well.

**Table 1:** Posterior parameter estimates for the spatial dynamic panel SAR specification and Poisson SEM specification

Coefficient	(i) Coefficient estimates			
	SAR		Poisson SEM	
	Mean	SD	Mean	SD
$\beta$	**0.944**	0.003	**0.003**	$< 10^{-5}$
$\sigma ^2$	**0.600**	0.007		
$R^2$	0.905		0.979	
*N*	99		99	
*T*	138		138	

	(ii) Average inclusion probability of $\varvec{W}(q_t)$			
	SAR		Poisson SEM	
Trade intensity	0.051		0.240	
Common borders	0.073		0.028	
Flight connections	0.766		0.692	
Free movement of people	0.109		0.039	

The model includes country fixed effects. Estimates in bold are statistically significant under a 95% confidence interval. Average posterior inclusion probabilities of $\varvec{W}(q_t)$ are reported for the period during which $\rho _t$ and $\lambda _t$ is significant under a 64% credible interval

Section (ii) of Table 1 contains the average posterior inclusion probability for the pre and post lockdown period of the four spatial weight matrices under scrutiny. Our models indicate that the main channel of virus transmissions before restrictive actions were taken can be attributed to international flight passengers and to a lesser degree to treaties guaranteeing free movement of people. However, international trade and common land borders played a comparatively minor role. Also, our models suggest that for the period after most countries entered a certain form of lockdown, neither of the four transmission channels predominantly explains the further spreading.

Turning attention to Fig. 2, panel (a) reveals a more differentiated picture of spatial spillovers in the SAR specification. The top panel contains the daily posterior inclusion probabilities of the four spatial weight matrices under scrutiny. The bottom panel depicts the smoothed daily posterior median estimate for the spatial autoregressive parameter $\rho _t$.

First, the daily estimates of spatial dependence confirm that initially spatial spillovers played a key and also statistically significant role in virus transmission: at the end of February only 4 countries within the sample (and only Italy within the EU) introduced flight suspension and $\rho _t$ is significant. After March 11th 2020, when the majority of European countries started introducing quarantine policies, closed their border crossings and reduced air travel, spatial autocorrelation becomes insignificant. Interestingly, in the mid of March, the spatial autoregressive parameter even becomes negative for a rather short period. One explanation for the short-term negative degree of spatial autocorrelation could be that some countries employed particularly tight travel bans to regions with high infection rates in order to reduce own-country virus transmission, resulting in dissimilarities (negative spatial autocorrelation) among spatial units. By March 24th, when over 3.5 billion people were living in some form of quarantine, spatial spillovers have become insignificant.

Second, the key role of flight travel is revealed to be only of importance in the first two months of our sample. Coupled with the suspension of international airline traffic in the beginning of March, the posterior importance of flight connections across all country significantly decreases. This is accompanied by a slight increase in the posterior importance of other measures of neighbourhood, particularly free movement of people within the EU.

The results of the Poisson SEM specification in Fig. 2, panel (b) largely confirm our findings from the SAR model. Spatial dependence markedly decreased after lockdown was implemented in the majority of countries and stayed insignificant in the following months. Furthermore, flight travel was the most significant spatial transmission channel between countries. The wider error margins and comparatively higher spatial dependence parameters are a direct result of the fundamental differences between the SAR and Poisson SEM specifications.

Overall, we find that spatial dependence notably decreased over time as countries elected to implement regional and global movement restrictions in the form of border closures, flight suspensions and even complete lockdowns. Additionally, air travel connections played a particularly important role in the early stages of the virus, suggesting that the shutdown of flight connections was indeed an important measure in mitigating early transmissions.

## Concluding remarks

We examine the virtues of spatial econometric specifications to study the spread of the recent coronavirus pandemic. Our results indicate that cross-country spatial spillover processes, specifically via international flight connections, played a particularly important role in the early stages of the virus spread. When countries began restricting airline traffic, the relative importance of flight connections as well as spatial autocorrelation decreased. Overall, our results imply that the shutdown of international airports and border closures were important policies to prevent further spillovers across countries. Moreover, the estimated spatial dependence structures seem to trace the process of the virus spread very well. Recent spatial econometric methods thus appear useful tools to model the global spread of coronavirus.

## Footnotes

1 For details on the MCMC estimation algorithm, see LeSage et al. (2007). R codes for both specifications used are available from the authors upon request.

2 Work by Jaya and Folmer (2020) or O’Hara and Kotze (2010), among several others, make use of spatial statistical approaches as a means to model the spread of diseases. Spatial statistical frameworks (see, for example, Blangiardo and Cameletti 2015) typically treat spatial spillover processes rather differently than spatial econometric approaches. Thorough discussions on the similarities and differences between spatial statistical approaches and spatial econometrics can be found in Bivand et al. (2014, (2015), or Gómez-Rubio et al. (2014).

3 In this study we make use of official and daily available COVID-19 infection data. It is, however, worth noting that underreported infections might severely affect the results. For a discussion and potential ways of correcting underreported infection numbers, see, among others, Jagodnik et al. (2020) or Krantz and Rao (2020).

4 Several checks using alternative ways to construct the spatial weight matrices confirm the robustness of the results.

5 It is worth noting that that the standard choice of an inverted gamma prior for the error nuisance might arguably severely affect the results (see, for example, Gelman 2006; Simpson et al. 2017). The penalised complexity (PC) prior advocated by Simpson et al. (2017) is a popular alternative frequently employed in the spatial statistics literature. Several model runs using alternative hyperparameters for the PC prior confirmed the robustness of our results. R codes as well as robustness checks using these alternative prior setups are available from the authors upon request.

6 *R* code for the estimation and datasets are available from the authors upon request.

7 Convergence of the sampler was checked using the diagnostics by Geweke (1992).

## References

[CR1] Anselin L (2013). Spatial Econometrics: Methods and Models.

[CR2] Bivand RS, Gómez-Rubio V, Rue H (2014). Approximate Bayesian inference for spatial econometrics models. Spat. Stat..

[CR3] Bivand RS, Gómez-Rubio V, Rue H (2015). Spatial data analysis with R-INLA with some extensions. J. Stat. Softw..

[CR4] Blangiardo M, Cameletti M (2015). Spatial and Spatio-Temporal Bayesian Models with R-INLA.

[CR5] Chagas AL, Carlos RA, Almeida A (2016). A spatial difference-in-differences analysis of the impact of sugarcane production on respiratory diseases. Reg. Sci. Urban Econ..

[CR6] Dong E, Du H, Gardner L (2020). An interactive web-based dashboard to track COVID-19 in real time. Lancet Infect. Dis..

[CR7] Emch M, Root ED, Giebultowicz S, Ali M, Perez-Heydrich C, Yunus M (2012). Integration of spatial and social network analysis in disease transmission studies. Ann. Assoc. Am. Geogr..

[CR8] Fischer MM, LeSage JP (2015). A Bayesian space-time approach to identifying and interpreting regional convergence clubs in Europe. Pap. Reg. Sci..

[CR9] Fischer MM, Scherngell T, Jansenberger E (2006). The geography of knowledge spillovers between high-technology firms in Europe: evidence from a spatial interaction modelling perspective. Geogr. Anal..

[CR10] Frühwirth-Schnatter S, Frühwirth R, Held L, Rue H (2009). Improved auxiliary mixture sampling for hierarchical models of non-Gaussian data. Stat. Comput..

[CR11] Gelman A (2006). Prior distributions for variance parameters in hierarchical models (comment on article by Browne and Draper). Bayesian Anal..

[CR12] Geweke J, Bernardo JM, Berger JO, Dawid AP, Smith AFM (1992). Evaluating the accuracy of sampling-based approaches to the calculation of posterior moments. Bayesian Statistics.

[CR13] Gómez-Rubio V, Bivand RS, Rue H, Fischer MM, Nijkamp P (2014). Spatial models using Laplace approximation methods. Handbook of Regional Science.

[CR14] Jagodnik, K., Ray, F., Giorgi, F.M., Lachmann, A.: Correcting under-reported COVID-19 case numbers: estimating the true scale of the pandemic. Preprint medRvix (2020)

[CR15] Jaya IGNM, Folmer H (2020). Bayesian spatiotemporal mapping of relative dengue disease risk in Bandung, Indonesia. J. Geogr. Syst..

[CR16] Krantz SG, Rao ASRS (2020). Level of underreporting including underdiagnosis before the first peak of COVID-19 in various countries: preliminary retrospective results based on wavelets and deterministic modeling. Infect. Control Hosp. Epidemiol..

[CR17] Krisztin T, Fischer MM (2015). The gravity model for international trade: specification and estimation issues. Spat. Econ. Anal..

[CR18] LeSage JP, Pace RK (2009). Introduction to Spatial Econometrics.

[CR19] LeSage JP, Chih YY (2018). A matrix exponential spatial panel model with heterogeneous coefficients. Geogr. Anal..

[CR20] LeSage JP, Fischer MM, Scherngell T (2007). Knowledge spillovers across Europe: evidence from a Poisson spatial interaction model with spatial effects. Pap. Reg. Sci..

[CR21] O’Hara R, Kotze J (2010). Do not log-transform count data. Methods Ecol. Evol..

[CR22] Piribauer P, Crespo Cuaresma J (2016). Bayesian variable selection in spatial autoregressive models. Spat. Econ. Anal..

[CR23] Simpson D, Rue H, Riebler A, Martins TG, Sørbye SH (2017). Penalising model component complexity: a principled, practical approach to constructing priors. Stat. Sci..

[CR24] Wang H, Du Z, Wang X, Liu Y, Yuan Z, Liu Y, Xue F (2015). Detecting the association between meteorological factors and hand, foot, and mouth disease using spatial panel data models. Int. J. Infect. Dis..

[CR25] Zoller HG (2004). Spatial Econometrics and Spatial Statistics.

